# Computed tomographic measurements of pancreatic thickness in clinically normal dogs

**DOI:** 10.3389/fvets.2023.1254672

**Published:** 2023-11-02

**Authors:** Yoojin An, Sungsoo Kim, Danbee Kwon, Kichang Lee, Hakyoung Yoon

**Affiliations:** ^1^Department of Veterinary Medical Imaging, College of Veterinary Medicine, Jeonbuk National University, Iksan-si, Jeollabuk-do, Republic of Korea; ^2^VIP Animal Medical Center, Seoul, Republic of Korea; ^3^Bundang Leaders Animal Medical Center, Seongnam-si, Gyeonggi-do, Republic of Korea

**Keywords:** pancreas, size, CT, canine, dimension

## Abstract

Pancreatic thickness is an indicator for evaluating pancreatic diseases. The transverse and cross-sectional pancreatic thickness observed on computed tomography (CT) may differ. This study aimed to provide a normal reference range for pancreatic thickness on the transverse plane based on body weight (BW) and assess pancreatic thickness to aorta (P/Ao) ratio. In addition, we aimed to establish the normal short and long dimensions of the pancreas based on cross-sectional image through the long axis of the pancreas using multiplanar reconstruction (MPR). The short dimension to aorta (S/Ao) and long dimension to aorta (L/Ao) ratios were also established in clinically normal dogs. The pancreatic thickness was measured using CT results of 205 clinically normal dogs. The pancreatic thickness on the transverse plane and the short and long dimensions in the cross-sectional image of the pancreas were measured using MPR. The diameter of the Ao was measured on the transverse plane and the P/Ao, S/Ao, and L/Ao ratios were calculated. Our study showed that the mean normal pancreatic thicknesses (mean ± standard deviation [SD]) of the pancreatic body, left and right lobe in the transverse plane were 10.92 ± 2.54 mm, 8.92 ± 2.26 mm and 9.96 ± 2.24 mm, respectively. The P/Ao ratios of the pancreatic body, left and right lobes were 1.85 ± 0.33, 1.50 ± 0.27 and 1.68 ± 0.29, respectively. The mean short dimension (mean ± SD) in the cross-sectional image of the pancreatic body, left and right lobe were 8.98 ± 1.97 mm, 7.99 ± 1.89 mm and 8.76 ± 2.03 mm, respectively. In conclusion, pancreatic thickness increased with BW, while the P/Ao, S/Ao, and L/Ao ratios could be used regardless of BW.

## 1. Introduction

Pancreatic diseases are relatively common in dogs and can present with a wide variety of clinical signs (1, 2). Although histopathology is the only definitive diagnosis for pancreatic diseases (3), other examinations, such as routine laboratory analysis, specific pancreatic enzyme assays, and diagnostic imaging, can also be helpful (1, 2).

Among diagnostic imaging modalities such as radiography, ultrasonography (US), and computed tomography (CT), US remains the primary modality for pancreatic evaluation and can be performed quickly in veterinary medicine (4, 5). Additionally, US can be used to evaluate various parts of the pancreas, including pancreatic thickness, which is necessary during pancreatic evaluation (6, 7). Some US studies have evaluated the normal reference range of pancreatic thickness of dogs and cats (8, 9) and have reported that pancreatic thickness increases with body weight (BW) (8), and can be used to assess pancreatic thikness using US.

Computed tomography (CT) is also considered to be a useful diagnostic imaging modality for evaluating the pancreas and can compensate for some of the limitations of the US (10–12). Several studies have described the appearance, normal vascular and parenchymal anatomy, pancreatic perfusion and enhancement pattern of the pancreas of dogs and cats using CT (11–19). There is also a study wherein the height, width, and length of the pancreas in nine normal beagle dogs was measured (14). The pancreas has an amorphous shape and lies at various positions, and its shape in the transverse plane on CT may not accurately represent its true thickness (8). Notably, since CT can use different planes, a more accurate thickness can be determined using a cross-section of the pancreas can be assessed.

Therefore, the purposes of this study were as follows: (1) to establish a normal reference range of pancreatic thickness on the transverse plane, (2) to obtain the short and long dimensions in the cross-sectional image of the long axis of the pancreas using MPR, and (3) to set ratios that can be applied regardless of body weight.

## 2. Materials and methods

### 2.1. Animals

In this retrospective and multicenter study, a total of 429 CT images and medical records from 2019 to 2022 were collected from three hospitals (Jeonbuk National University Animal Medical Center, Bundang Leaders Animal Medical Center, VIP animal Medical Center). The inclusion criteria were as follows: no evidence of gastrointestinal tract disease based on history, clinical signs, physical examination, laboratory blood tests, US, and CT images, and dogs with 4–6/9 body condition scores (BCS). Dogs with ascites, abdominal tumors, or abnormalities in the pancreatic parenchyma and peripancreatic region, including surrounding fat, on US or CT were excluded. Dogs with obesity (> 7/9 BCS) were also excluded. In total, 205 CT images were included, and the animals were classified into four groups according to body weight (BW), group A (*n* = 104): ≤5 kg; group B (*n* = 68): >5 kg, ≤10 kg; group C (*n* = 17): >10 kg, ≤15 kg; group D (*n* = 16): >15 kg, ≤ 30 kg. This study was approved by the Institutional Animal Care and Use Committee of the Jeonbuk National University, Iksan-si, Jeollabuk-do, Republic of Korea (approval no. NON2022-054).

### 2.2. CT scan protocol and measurements

All CT images were reviewed in the delayed phase and RadiAnt DICOM viewer (Pozana, Poland) was used.

The CT images were acquired using three scanners: 1) Brivo CT 385(GE Medical System CO., LTD, Beijing, China) using 110–120 kVp, 80–110 mAs, 1.0–1.5 mm slice thickness, 0.5 mm reconstructed slice thickness, 1.0 s rotation time, and 0.938 collimation beam pitch, 2) the Brivo CT 385 (GE Medical System CO., LTD, Beijing, China) using 110–120 kVp, 80–120 mAs, 1.0–1.25 mm slice thickness, 0.5 mm reconstructed slice thickness, 1.0 s rotation time, and 0.938 collimation beam pitch, and 3) the Alexion 16 (Toshiba Medical Systems Co Ltd., Otowara, 111 Japan) using 120 kVp, 110–200 mAs, 1.0–1.25 mm slice thickness, 0.75 s rotation time, 0.5 mm reconstructed slice thickness, and 1.375 collimation beam pitch. The CTs were performed with the dogs in sternal recumbency. All transverse plane images were obtained in a head-to-tail direction and perpendicular to the spine. Iohexol (Omnipaque, 600–750 mg/kg; GE Healthcare, Ireland) was used as a contrast medium and administered via the cephalic vein. Post-contrast images were obtained 120–150 s after contrast administration. All CT images were reviewed in the abdominal soft tissue window [window level = 40–45 Hounsfield units (HU); window width = 400–450,114 HU].

**Figure 1:**
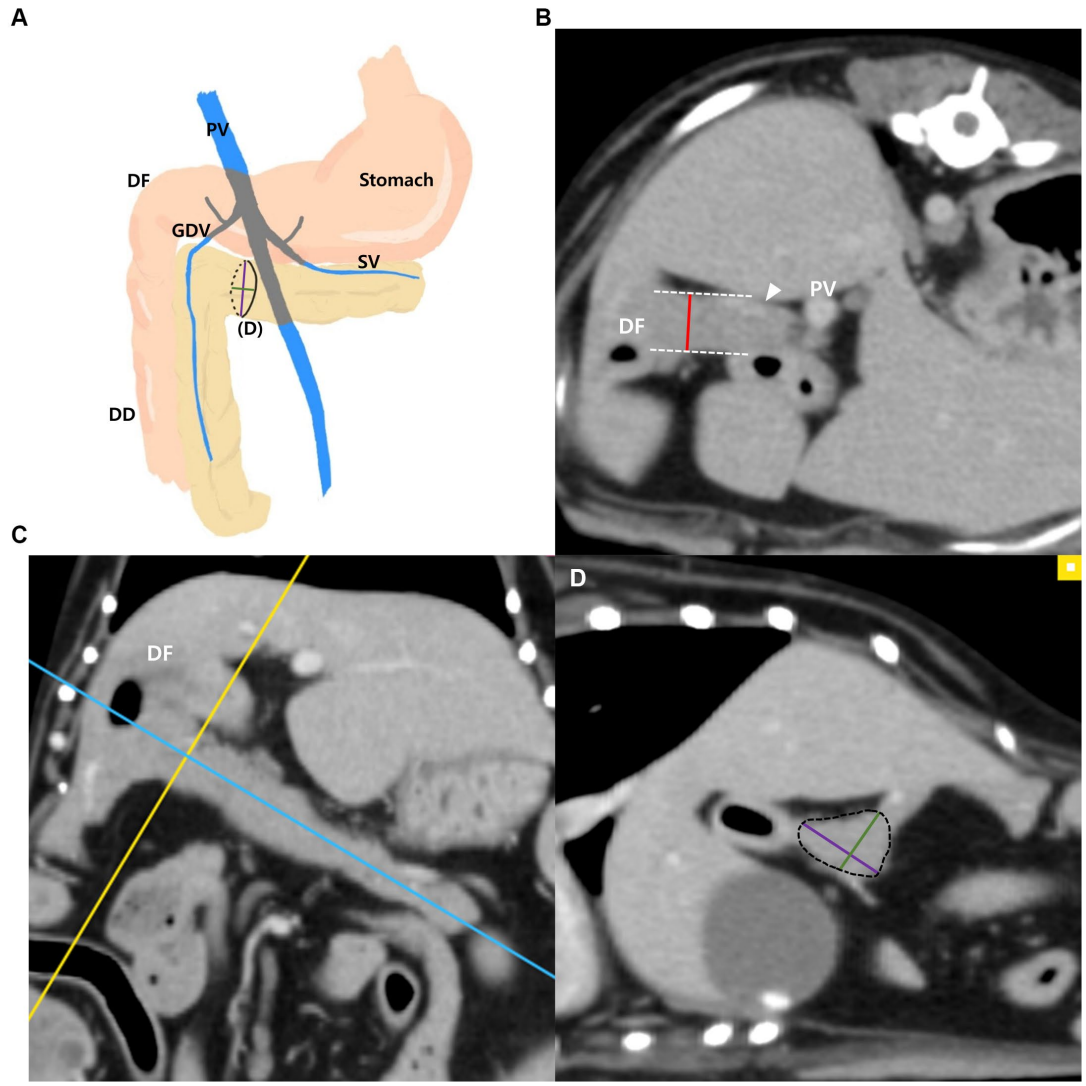
Measurements of the pancreatic body. Schematic illustration of pancreas **(A)**, transverse plane **(B)**, dorsal plane **(C)** and oblique sagittal MRP plane **(D)**. The thickness of the pancreatic body was measured at the thickest location adjacent to the duodenal flexure (DF) **(B)**. On the dorsal plane **(C)**, long axis (blue line) of pancreas was set to be parallel to the pancreas and through the middle. MPR was performed perpendicular (yellow line) to the long axis (blue line) of the pancreatic body connecting to the left lobe at the thickest part. In the oblique sagittal MPR plane **(D)**, which is cross sectional image at the yellow line, short and long dimensions perpendicular to each other were measured at their longest length (Green, short dimension; Purple, long dimension). PV, portal vein; DF, duodenal flexure; DD, descending duodenum; GDV, gastroduodenal vein (arrowhead); SV, splenic vein.

The measurements of the pancreatic body (Figures 1A–D) were taken as follows: the thickness of the pancreatic body was measured at the thickest location in the region adjacent to the duodenal flexure on the transverse plane (Figure 1B). Additionally, in the thickest part, MPR was performed perpendicular to the long axis of the pancreatic body that connecting to the left pancreatic lobe on the dorsal plane (Figure 1C). The long axis of the pancreas was set to be parallel to the midline of the pancreas. In the cross-sectional image (oblique sagittal MPR plane) of the long axis of the pancreatic body, two dimensions perpendicular to each other were measured (Figure 1D). Two dimensions were measured at the longest location and the shorter dimension was called the “short dimension,” and the longer dimension was called the “long dimension” in this study (Figure 1D). The vessels adjacent to or overlapping the pancreas were included in the measurements.

**Figure 2:**
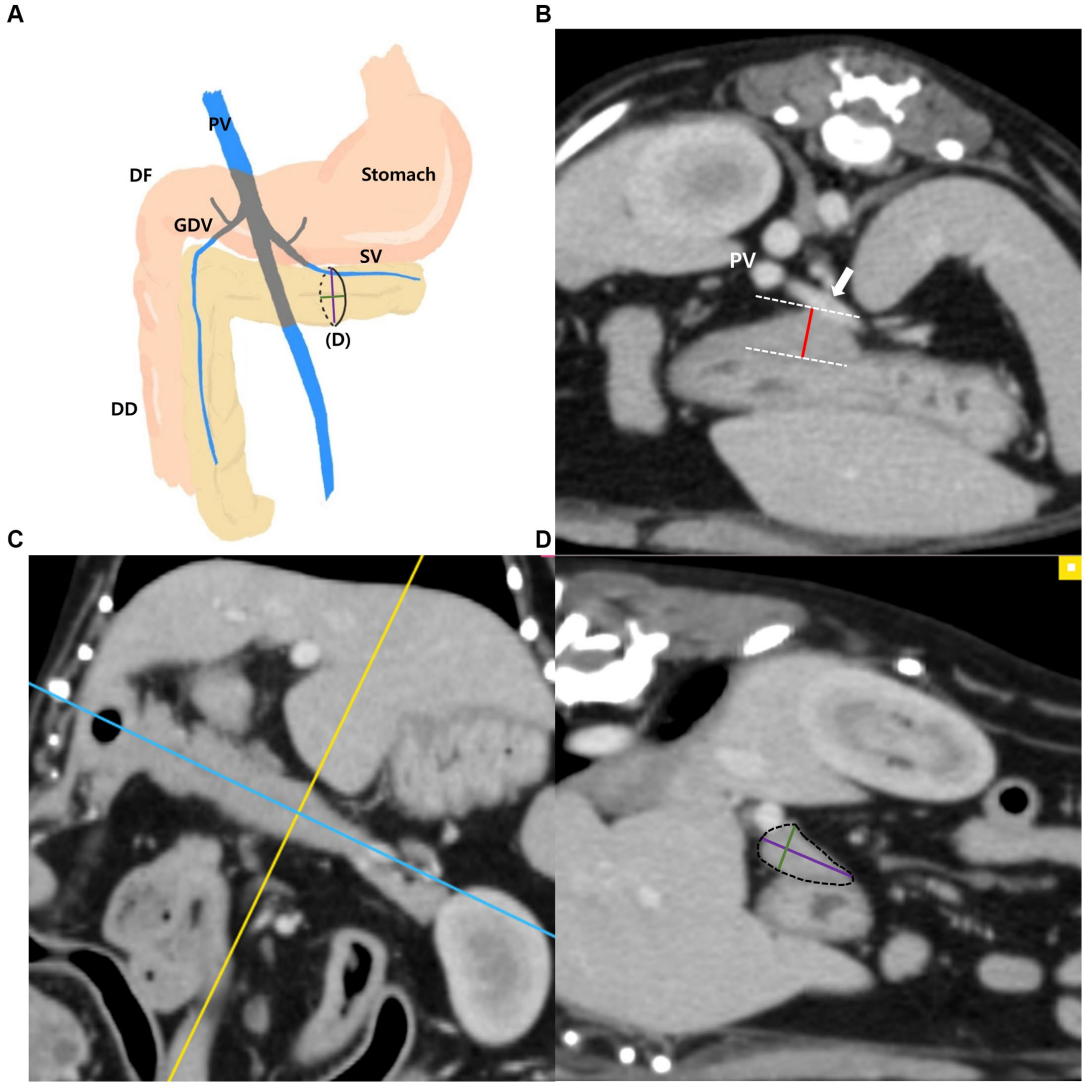
Measurements of the left pancreatic lobe. Schematic illustration of pancreas **(A)**, transverse plane **(B)**, dorsal plane **(C)** and oblique sagittal MPR plane **(D)**. The thickness of the left lobe was measured at the thickest location along the length of the left lobe (white arrow, splenic vein) **(B)**. On the dorsal plane **(C)**, long axis (blue line) of pancreas was set to be parallel to the pancreas and through the middle. MPR was performed perpendicular (yellow line) to the long axis (blue line) of the left lobe at the thickest part. In the oblique sagittal MPR plane **(D)** which is cross sectional image at the yellow line, short and long dimensions perpendicular to each other were measured at their longest length (Green, short dimension; Purple, long dimension). PV, portal vein; DF, duodenal flexure; DD, descending duodenum; GDV, gastroduodenal vein; SV, splenic vein (white arrow).

The measurements of the left pancreatic lobe (Figures 2A–D) were taken as follows: the left lobe was measured at the thickest location along the length of the left lobe extending adjacent to the splenic vein on the transverse plane (Figure 2B). Additionally, in the thickest part, MPR was performed perpendicular to the long axis of the left lobe on the dorsal plane (Figure 2C). The long axis of the pancreas was set to be parallel to the pancreas and through the middle. In the cross-sectional image (oblique sagittal MPR plane) of the long axis of the left lobe, two dimensions perpendicular to each other were measured (Figure 2D). Two dimensions were measured at the longest location and the shorter dimension was called the “short dimension” and the longer dimension was called the “long dimension,” respectively (Figure 2D).

**Figure 3:**
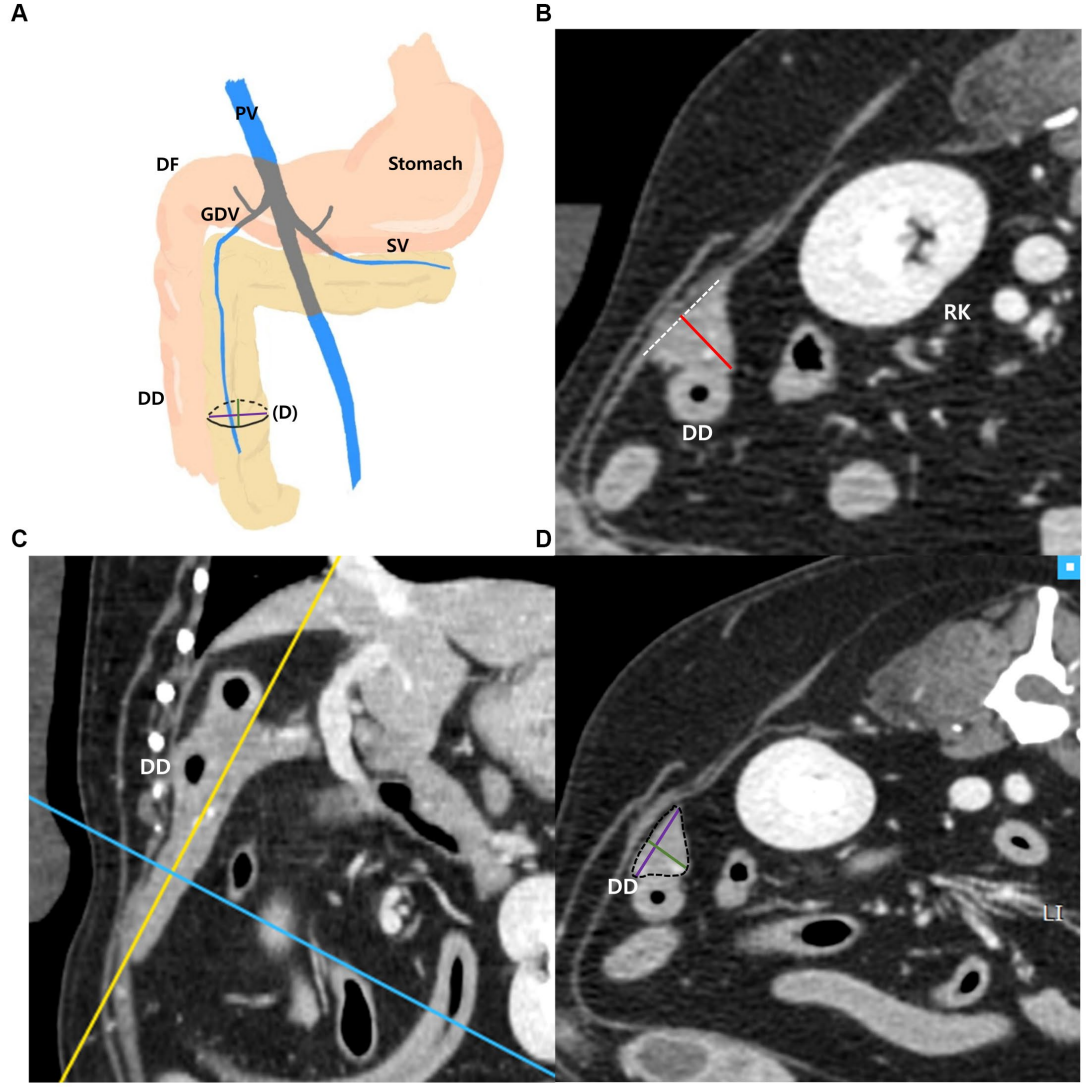
Measurements of the right pancreatic lobe. Schematic illustration of pancreas **(A)**, transverse plane **(B)**, dorsal plane **(C)** and oblique transverse MPR plane **(D)**. The thickness of the right lobe was measured at the thickest location in the region that runs along the descending duodenum (DD) **(B)**. On the dorsal plane **(C)**, long axis (yellow line) of pancreas was set to be parallel to the pancreas and through the middle. MPR was performed perpendicular (blue line) to the long axis (yellow line) of the right lobe at the thickest part. In the oblique transverse MPR plane **(D)** which is cross sectional image at the blue line, short and long dimensions perpendicular to each other were measured at their longest length (Green, short dimension; Purple, long dimension). PV, portal vein; DF, duodenal flexure; DD, descending duodenum; GDV, gastroduodenal vein; SV, splenic vein; RK, right kidney.

The measurements of the right pancreatic lobe were taken as follows (Figures 3A–D): the right lobe was measured at the thickest location in the region that runs along the descending duodenum on the transverse plane (Figure 3B). Additionally, in the thickest part, MPR was performed perpendicular to the long axis of the right lobe on the dorsal plane (Figure 3C). The long axis of the pancreas was set to be parallel to the pancreas and through the middle. In the cross-sectional image (oblique transverse MPR plane) of the long axis of the right lobe, two dimensions perpendicular to each other were measured (Figure 3D). Two dimensions were measured at the longest location and the shorter dimension was called the “short dimension,” and the longer dimension was called the “long dimension,” respectively (Figure 3D).

**Figure 4:**
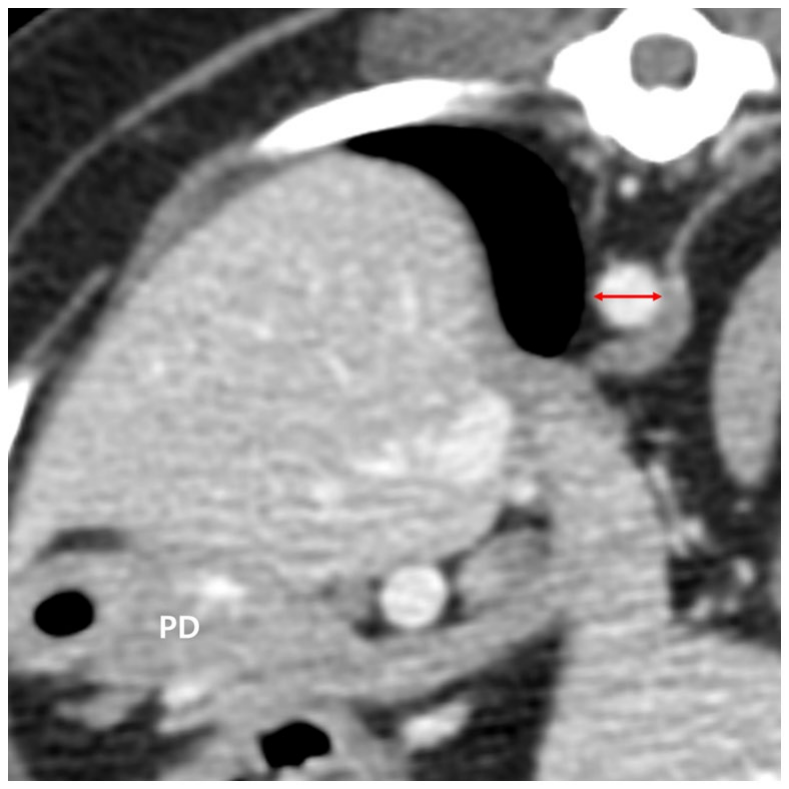
The diameter of the aorta (red double arrow) was measured in the transverse plane horizontally at the level where the pancreatic body (PD) was measured. PD, pancreatic body.

The diameter of the aorta (Ao) was measured horizontally (right lateral to left lateral) at the level where the pancreatic body was measured on the transverse plane (Figure 4). Additionally, the ratio of the pancreatic thickness measured on transverse plane to aorta (P/Ao), short dimension to aorta (S/Ao) ratio, and long dimension to aorta (L/Ao) ratio were calculated. The short and long dimension are measurements taken in a cross-sectional image (oblique sagittal or transverse MPR plane) through the long axis of the pancreas.

All measurements were assessed and recorded in duplicates by observer A and observer B (both second year veterinary radiology residents in the Veterinary Medical Imaging Department of the Teaching Hospital of Jeonbuk National University).

### 2.3. Statistics

Linear regression analysis was performed to evaluate the correlation between BW and pancreatic thickness. Analysis of variance (ANOVA) ANOVA was used to evaluate the differences in pancreatic thickness between the BW groups. Additionally, ANOVA was also used to evaluate differences in the P/A, S/Ao, and L/Ao ratios of each pancreatic lobe among BW groups. Pearson’s correlation analysis was used to assess the correlation between pancreatic thickness and age, and an independent t-test was used to evaluate the differences between pancreatic thickness and sex. Statistical analyzes were performed using IMB SPSS software (version 27.0; Chicago, IL, United States) for all analyzes, and values of *p* < 0.001 or *p* < 0.05 were considered statistically significant. Intra-and interobserver reliabilities for all measurements were evaluated using the absolute agreement-type intraclass correlation coefficient (ICC) with a 95% confidence interval (CI).

## 3. Results

### 3.1. Animals

Among a total of 205 dogs, 76 were neutered females, 18 were intact females, 96 were neutered males, and 15 were intact males. The mean age (mean ± standard deviation [SD]) of the dogs was 8.66 ± 3.57 years and the mean BW (mean ± standard deviation [SD]) was 6.78 ± 5.32 kg. The breeds of all 205 dogs were as follow: Maltese (54), Poodle (28), Mixed (21), Pomeranian (13), Shih tzu (11), Cocker spaniel (13), Yorkshire terrier (9), Chihuahua (8), Schnauzer (7), Dachshund (6), Pekingese (5), Spitz (4), Jindo (3), Pug (2), Italian greyhound (2), French bulldog (2), Boston terrier (2), Bichon fries (2), Beagle (2), Shiba inu (1), Papillon (1), King Charles spaniel (1), Samoyed (1), Standard poodle (1), Chowchow (1), Shetland sheepdog (1), Golden retriever (1), Labrador retriever (1), Shar pei (1), Dalmatian (1).

The most common reasons for CT scans were musculoskeletal disorders and surgical planning (*n* = 42 [22.2%]), nasal cavity disorders (*n* = 29 [15.3%]), ear disorders (*n* = 16 [8.5%]), urinary tract problems (*n* = 12 [6.3%]), screening tests before MRI (*n* = 12 [6.3%]), disorders of the salivary gland (*n* = 10, [5.3%]), respiratory problems (*n* = 8 [4.2%]) and health check-up (*n* = 8 [4.2%]). Other reasons included reproductive system disorders, foreign body, hernia, ophthalmic disorders, evaluation of mammary gland tumor, and other problems.

The number of cases acquired with each of the CT scanners are as follows: 1) 106 cases; 2) 56 cases; 3) 43 cases.

### 3.2. The pancreatic thickness on the transverse plane and correlation with BW, and P/Ao ratios

**Figure 5:**
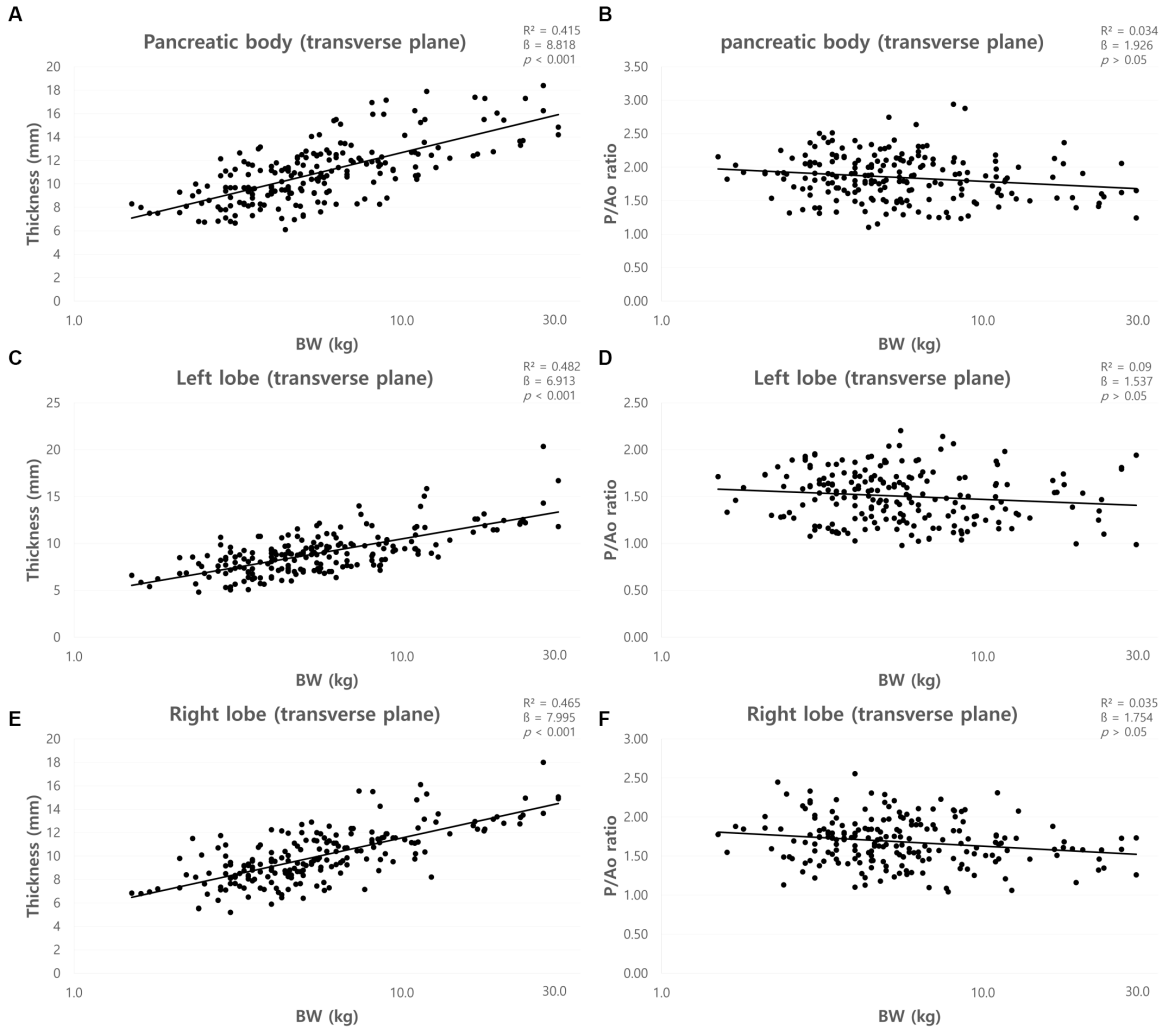
Correlation between pancreatic thickness and body weight (BW) and between pancreatic thickness to Ao (P/Ao) ratios and BW. The thickness of pancreatic body **(A)**, left lobe **(C)**, and right lobe **(E)** measured on the transverse plane. All pancreatic thickness showed a linear positive correlation with BW. P/Ao ratio of the pancreatic body **(B)**, P/Ao ratio of the left lobe **(D)**, and P/Ao ratio of the right lobe **(F)**. P/Ao ratios of pancreas showed no correlation with BW. BW, body weight; P/Ao, pancreatic thickness measured on the transverse plane to aorta.

The mean total pancreatic thickness (mean ± standard deviation [SD]) and the pancreatic thickness for each BW group were summarized in Table 1. The thickness of the pancreas increased with BW, and ANOVA showed a significant difference between the BW groups (*p* < 0.05). Furthermore, there was a positive correlation between BW and the pancreatic thickness (*p* < 0.001) (Figures 5A,C,E).

**Table 1:** The pancreatic thickness on the transverse plane.

		Mean ± SD (mm) (95% CI)		
		Body	Lt	Rt
BW	Group A; ≤5 kg (*n* = 104)	9.43 ± 1.64 (9.12–9.76)	7.80 ± 1.51 (7.51–8.10)	8.66 ± 1.56 (8.36–8.97)
	Group B; >5 kg, ≤10 kg (*n* = 68)	11.65 ± 2.11 (11.15–12.17)	9.18 ± 1.58 (8.80–9.57)	10.57 ± 1.67 (10.17–10.98)
	Group C; >10 kg, ≤15 kg (*n* = 17)	13.11 ± 2.10 (12.01–14.20)	10.89 ± 2.20 (9.76–12.02)	11.98 ± 2.11 (10.90–13.07)
	Group D; >15 kg, ≤ 30 kg (*n* = 16)	15.05 ± 1.78 (14.10–15.99)	13.06 ± 2.35 (11.80–14.31)	13.60 ± 1.50 (12.81–14.40)
	Total (*n* = 205)	10.92 ± 2.54 (10.57–11.27)	8.92 ± 2.26 (8.62–9.24)	9.96 ± 2.24 (9.65–10.27)
Difference between BW groups		A vs. B vs. C vs. D^*^	A vs. B vs. C vs. D^*^	A vs. B vs. C vs. D^*^

SD, standard deviation; CI, confidence interval; Lt, left lobe; Rt, right lobe; *p* < 0.05^*^ were considered significant.

The mean P/Ao ratios were summarized in Table 2. ANOVA showed no significant difference between the BW groups (*p* > 0.05). Furthermore, there was no correlation between BW and P/Ao ratio of the pancreas (*p* > 0.05) (Figures 5B,D,F).

**Table 2:** The mean ratios of pancreatic measurement to aorta (Ao).

	Mean ± SD (95% CI)		
	Body	Lt	Rt
P/Ao ratio	1.85 ± 0.33 (1.80–1.89)	1.50 ± 0.27 (1.47–1.54)	1.68 ± 0.29 (1.64–1.72)
S/Ao ratio	1.52 ± 0.28 (1.48–1.56)	1.36 ± 0.25 (1.32–1.39)	1.48 ± 0.27 (1.44–1.52)
L/Ao ratio	1.97 ± 0.38 (1.92–2.02)	1.83 ± 0.39 (1.78–1.88)	1.90 ± 0.38 (1.85–1.95)

P/Ao, pancreatic thickness measured on the transverse plane to aorta; S/Ao, short dimension to aorta; L/Ao, long dimension to aorta; SD, standard deviation; CI, confidence interval; Lt, left lobe; Rt, right lobe.

### 3.3. The short dimensions of the pancreas in the cross-sectional image of the long axis of the pancreas using MPR and correlation with BW, and S/Ao ratios

**Figure 6:**
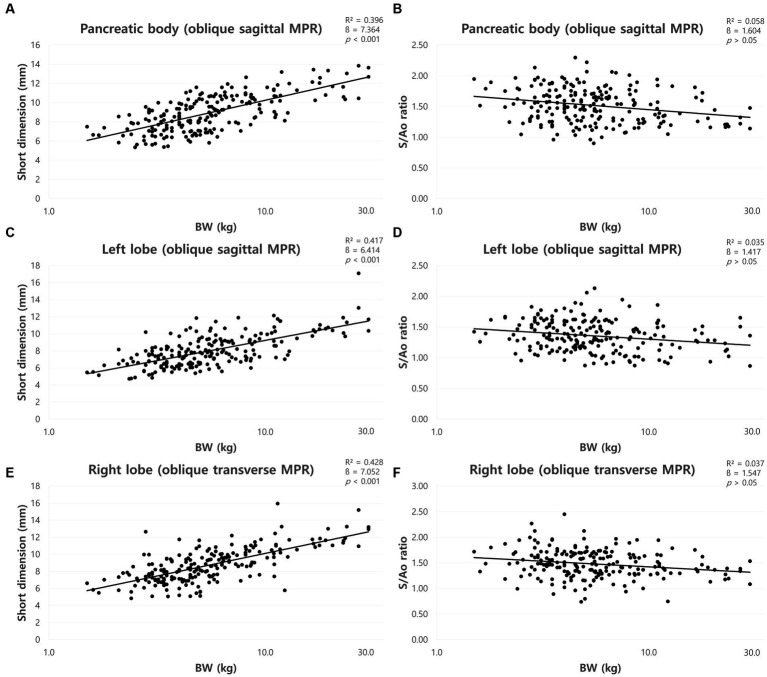
Correlation between short dimensions of pancreas and body weight (BW) and between short dimension to Ao (S/Ao) ratios and BW. Short dimension of pancreatic body **(A)**, left lobe **(C)**, and right lobe **(E)**. The short dimensions of the pancreas showed a linear positive correlation with BW. S/Ao ratio of pancreatic body **(B)**, left lobe **(D)**, and right lobe **(F)**. S/Ao ratios of the pancreas showed no correlation with BW. BW, body weight; S/Ao, short dimension measured in the cross-sectional image to Ao.

The mean short dimensions (mean ± SD) of the pancreas and the short dimensions for each BW group were summarized in Table 3. The short dimensions of the pancreas increased with BW, and ANOVA showed a significant difference between the BW groups (*p* < 0.05). There was a positive correlation between BW and the short dimensions of the pancreas (*p* < 0.001) (Figures 6A,C,E).

**Table 3:** The short dimensions in the cross-sectional image of the long axis of the pancreas using MPR.

		Mean ± SD (mm) (95% CI)		
		Body	Lt	Rt
BW	Group A; ≤5 kg (*n* = 104)	7.87 ± 1.43 (7.60–8.16)	7.07 ± 1.38 (6.80–7.34)	7.63 ± 1.52 (7.34–7.93)
	Group B; >5 kg, ≤10 kg (*n* = 68)	9.44 ± 1.50 (9.01–9.81)	8.29 ± 1.35 (7.97–8.63)	9.20 ± 1.28 (8.90–9.52)
	Group C; >10 kg, ≤15 kg (*n* = 17)	10.49 ± 1.42 (9.77–11.23)	9.56 ± 1.62 (8.73–10.40)	10.49 ± 2.23 (9.34–11.65)
	Group D; >15 kg, ≤ 30 kg (*n* = 16)	12.06 ± 1.34 (11.35–12.78)	11.19 ± 1.78 (10.23–12.15)	12.06 ± 1.12 (11.46–12.66)
	Total (*n* = 205)	8.95 ± 1.97 (8.68–9.22)	7.99 ± 1.89 (7.73–8.25)	8.76 ± 2.03 (8.48–9.04)
Difference between BW groups		A vs. B vs. C vs. D^*^	A vs. B vs. C vs. D^*^	A vs. B vs. C vs. D^*^

SD, standard deviation; CI, confidence interval; BW, body weight; Lt, left lobe; Rt, right lobe; *p* < 0.05^*^ were considered significant.

The mean S/Ao ratios were summarized in Table 2. ANOVA showed no significant difference between the BW groups (*p* > 0.05). Furthermore, there was no correlation between BW and S/Ao ratio of the pancreas (*p* > 0.05) (Figures 6B,D,F).

### 3.4. The long dimensions of the pancreas in the cross-sectional image of the long axis of the pancreas using MPR and correlation with BW, and L/Ao ratios

**Figure 7:**
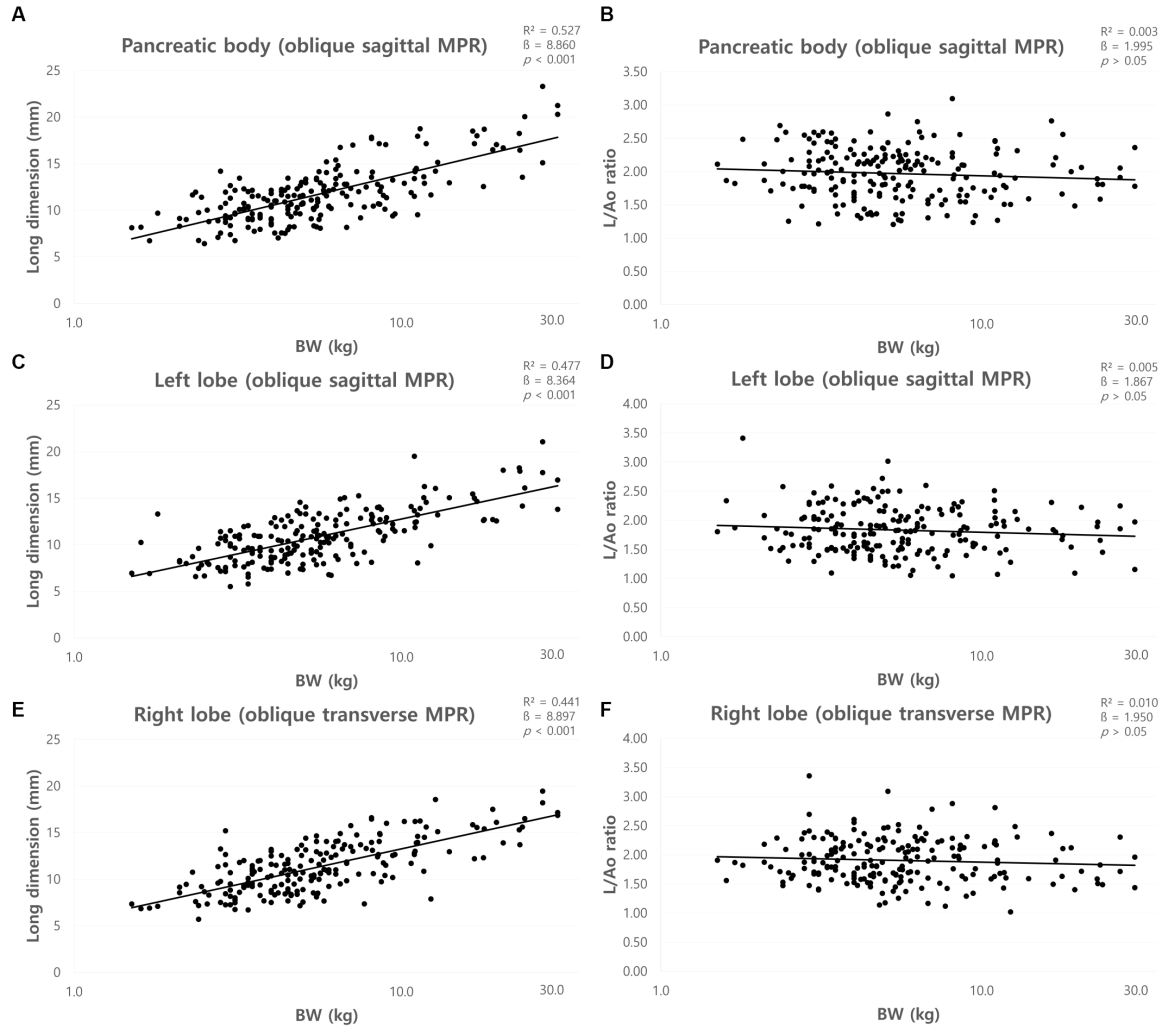
Correlation between long dimension of pancreas and body weight (BW) and between long dimensions to Ao (L/Ao) ratios and BW. Long dimension of pancreatic body **(A)**, left lobe **(C)**, and right lobe **(E)**. The long dimensions of the pancreas showed a linear positive correlation with BW. L/Ao ratio of pancreatic body **(B)**, left lobe **(D)**, and right lobe **(F)**. L/Ao ratios of the pancreas showed no correlation with BW. BW, body weight; L/Ao, long dimension measured in the cross-sectional image to Ao.

The mean long dimensions (mean ± SD) of the pancreas and the long dimensions for each BW group were summarized in Table 4. The long dimensions of the pancreas increased with BW, and ANOVA showed a significant difference between the BW groups (*p* < 0.05) except the dimensions of the right lobe between group C and D. There was a positive correlation between BW and long dimensions of the pancreas (*p* < 0.001) (Figures 7A,C,E).

**Table 4:** The long dimensions in the cross-sectional image of the long axis of the pancreas using MPR.

		Mean ± SD (mm) (95% CI)		
		Body	Lt	Rt
BW	Group A; ≤5 kg (*n* = 104)	10.02 ± 1.72 (9.69–10.36)	9.37 ± 1.91 (9.01–9.45)	9.64 ± 1.87 (9.28–10.02)
	Group B; >5 kg, ≤10 kg (*n* = 68)	12.19 ± 2.36 (11.63–12.77)	11.23 ± 2.02 (10.75–11.73)	12.05 ± 2.17 (11.53–12.58)
	Group C; >10 kg, ≤15 kg (*n* = 17)	13.89 ± 2.38 (12.67–15.12)	13.56 ± 2.60 (12.23–14.91)	13.92 ± 2.47 (12.65–15.19)
	Group D; >15 kg, ≤ 30 kg (*n* = 16)	17.63 ± 2.60 (16.24–19.01)	15.69 ± 2.41 (14.40–16.97)	15.69 ± 1.99 (14.63–16.75)
	Total (*n* = 205)	11.65 ± 2.99 (11.24–12.07)	10.84 ± 2.79 (10.45–11.22)	11.29 ± 2.81 (10.90–11.68)
Difference between BW groups		A vs. B vs. C vs. D^*^	A vs. B vs. C vs. D^*^	A vs. B vs. C^*^, A vs. D^*^, B vs. D^*^

SD, standard deviation; CI, confidence interval; BW, body weight; Lt, left lobe; Rt, right lobe; *p* < 0.05^*^ were considered significant.

The mean L/Ao ratios were summarized in Table 2. ANOVA showed no significant difference between the BW groups (*p* > 0.05). Furthermore, there was no correlation between BW and L/Ao ratio of the pancreas (*p* > 0.05) (Figures 7B,D,F).

### 3.5. The correlation between age and pancreatic thickness and significant differences in pancreatic thickness between sexes

The correlation between age and all pancreatic measurements was evaluated using Pearson’s correlation analysis, which showed no correlation (*p* > 0.05), except for the short and long dimension of the right lobe and short dimension of the left lobe. There was a weak positive correlation between age and short and long dimensions of right lobe and short dimension of left lobe in Pearson’s correlation analysis (*p* < 0.05).

There was no significant difference in pancreatic thickness between sexes in the independent *t*-test (*p* > 0.05). In addition, there were no significant differences in any of the measurements between the neutered and intact females or neutered and intact males in the independent *t*-test (*p* > 0.05).

### 3.6. Intra-and interobserver reliability

The measurements were performed in duplicates by observer A, and the median ICC showed excellent reliability for all measurements. The intra-observer reliabilities measured by ICC were all >0.962 (*p* < 0.001). All measurements were repeated by observer B, and the median ICC showed good to excellent reliability for all measurements. The interobserver reliabilities measured by ICC were all >0.848 (*p* < 0.001).

## 4. Discussion

This study established normal reference ranges for pancreatic measurements on CT in clinically normal dogs.

In a previous study that established reference ranges for normal pancreatic thickness using US in dogs (8), reference ranges for dog under 30 kg were smaller than those in our study using CT. This was partially consistent with a previous study in human medicine which compared normal US and CT biometry of the pancreatic segment and reported that the dimensions of the pancreas measured using CT were significantly larger than those measured using US (20). This difference was reported to be due to the inclusion of the splenic and superior mesenteric veins in the pancreatic diameter measured using CT (20–22). Our study may also have included adjacent or overlapping blood vessels which can cause discrepancies in pancreatic measurements using the US. Given these differences in values of US and CT, using the measurements from this study to assess pancreatic thickness on CT would be advantageous.

Pancreatic and peripancreatic anatomy, vascular and parenchymal enhancement in dogs using single-slice helical CT technology, and pancreatic measurements such as height, width, and length in nine beagle dogs were presented in a previous study (14). The pancreatic measurements in previous study were obtained from 9 beagle dogs with a mean body weight of 20 kg (14). In the present study, the reference ranges considering each BW groups were obtained from a larger sample. Since the evaluation of pancreatic thickness is important in pancreatic diseases (7), the normal reference ranges derived from this study can be applied differently according to BW.

The pancreas has an amorphous and almost triangular to rounded shape in cross-section through the long axis of the pancreas (7, 23). The position of the pancreas can shift with the dog’s posture (8), thus the pancreatic thickness measured on the transverse plane may differ from the actual thickness of the pancreas. Therefore, in this study, the short and long dimension of the pancreas were also obtained in the cross-sectional image through the long axis of the pancreas using MPR. However, there was no significant difference in the long dimension of the right lobe between group C and D. This is considered to be due to the small sample size of medium to large breed dogs. The short dimension was smaller than the pancreatic thicknesses measured on the transverse plane, which may be more indicative of the actual thickness of the pancreas. Thus, short dimensions can be used to assess pancreatic thickness more accurately on CT.

In this study, which included dogs with a relatively standard BCS, the thickness of the pancreas increased with BW, consistent with a previous study (8). Additionally, this study attempted to derive parameters that could be used to evaluate pancreatic thickness, regardless of BW. Several studies have used aortic diameter as an indicator of organ size (24–26). In a previous study, it was reported that the direction of change in the diameter of the aorta was larger from anterior to posterior (ventral to dorsal in dogs) than from the right lateral to the left lateral, and that the change in diameter decreased toward the abdominal aorta. Therefore, the aorta diameter was measured in the horizontal direction, where there is less diameter variation (27). In our study, the P/Ao ratios, S/Ao and L/Ao ratios were obtained using the aorta diameter. These ratios were obtained for the pancreatic body, left and right pancreatic lobe and confirmed by ANOVA that the parameters were constant, regardless of BW. Therefore, these values can be useful indicators, regardless of BW.

The thickness of the pancreas had no correlation with age, except for the short and long dimension of right lobe and short dimension of left lobe, which had a weak positive correlation with age. In previous studies, no significant correlation was found between age and pancreatic thickness in clinically normal dogs or cats (8, 28). In humans, it has been reported that as pancreatic fat volume increases with age, the pancreatic volume also increases (29).

There was no significant difference in pancreatic thickness between sexes. In humans, the pancreatic volume is greater in males than in females (20, 29, 30), which is considered to be due to anatomical differences caused by differences in body physique between males and females. However, unlike humans, the difference in body size between females and males is similar in dogs, and it was considered that there was no significant difference in pancreatic thickness between females and males.

Intra-and inter-observer reliability analyzes were performed to confirm the reliability of the measurements. The intra-and inter-class correlation coefficients were all above 0.84 for all values, indicating almost perfect agreement (31). Therefore, all the measured values and normal reference ranges derived in this study were considered reliable.

This study has a few limitations. The sample size for medium and large breed dogs is smaller than that of small breed dogs which should be improved in the further studies. Furthermore, because the pancreas is difficult to clearly divide into lobes anatomically, measurements may be slightly subjective. In particular, the pancreatic body is a curved segment that connects the left and right lobes, thus there may be variation in the measurement location. In this study, pancreatic body was measured at a defined location, which is the pancreatic body connecting the left lobe and is adjacent to the duodenal flexure. In addition, due to the retrospective nature of this study, the US data was not available for all patients. The thickness of the pancreas could not be compared between US and CT in the same patient; accordingly, the differences in measurements between the two modalities could not be directly compared, as in a previous study in humans. Finally, in this study, histopathology, which is a definitive diagnostic method for pancreatic disease (6, 32, 33) was not available in all patients. Thus, patients with subclinical pancreatic diseases could not be completely excluded.

In conclusion, this is the first study to establish a normal reference range for the pancreatic thickness on the transverse plane and the P/Ao ratio. Additionally, the short and long dimensions in the cross-sectional image of the long axis of the pancreas, S/Ao ratios, and L/Ao ratios were established in clinically normal dogs. This study showed that the thickness of the pancreas increases with BW in dogs with similar physiques; thus, it can be applied differently depending on BW, whereas the P/Ao, S/Ao, and L/Ao ratios can be used regardless of BW.

## Data availability statement

The raw data supporting the conclusions of this article will be made available by the authors, without undue reservation.

## Ethics statement

The animal studies were approved by Institutional Animal Care and Use Committee of the Jeonbuk National University, Iksan-si, Jeollabuk-do, Republic of Korea (approval no. NON2022-054). The studies were conducted in accordance with the local legislation and institutional requirements. Written informed consent was obtained from the owners for the participation of their animals in this study.

## Author contributions

YA: Conceptualization, Data curation, Formal analysis, Investigation, Methodology, Project administration, Writing – original draft, Writing – review & editing, Software. SK: Data curation, Formal analysis, Investigation, Writing – review & editing. DK: Data curation, Formal analysis, Investigation, Writing – review & editing. KL: Conceptualization, Data curation, Formal analysis, Methodology, Project administration, Supervision, Validation, Writing – review & editing. HY: Conceptualization, Data curation, Formal analysis, Investigation, Methodology, Project administration, Supervision, Validation, Writing – original draft, Writing – review & editing.

## Abbreviations

Ao: abdominal aorta
BW: body weight
SD: standard deviation
US: ultrasonography
CT: computed tomography
MPR: multiplanar reconstruction
P/Ao ratio: pancreatic thickness measured on the transverse plane to aorta ratio
S/Ao ratio: short dimension to Ao ratio
L/Ao ratio: long dimension to Ao ratio
